# A robust and efficient automatic method to segment maize FASGA stained stem cross section images to accurately quantify histological profile

**DOI:** 10.1186/s13007-022-00957-0

**Published:** 2022-11-24

**Authors:** P.-L. Lopez-Marnet, S. Guillaume, V. Méchin, M. Reymond

**Affiliations:** 1 Université Paris-Saclay, INRAE, AgroParisTech, Institut Jean-Pierre Bourgin (IJPB), 78000 Versailles, France; 2 Ecole Doctorale Numéro 581 : ABIES, AgroParisTech, Université Paris-Saclay, 19 Av du Maine, 75732 Paris Cedex 15, France

**Keywords:** Image analysis, Automatic workflow, Segmentation, Fasga stained cross section, Plant tissues, Quantitative histology, H, S and V dimensions, Universal tool

## Abstract

**Background:**

Grasses internodes are made of distinct tissues such as vascular bundles, epidermis, rind and pith. The histology of grasses stem was largely revisited recently taking advantage of the development of microscopy combined with the development of computer-automated image analysis workflows. However, the diversity and complexity of the histological profile complicates quantification. Accurate and automated analysis of histological images thus remains challenging.

**Results:**

Herein, we present a workflow that automatically segments maize internode cross section images into 40 distinct tissues: two tissues in the epidermis, 19 tissues in the rind, 14 tissues in the pith and 5 tissues in the bundles. This level of segmentation is achieved by combining the Hue, Saturation and Value properties of each pixel and the location of each pixel in FASGA stained cross sectiona. This workflow is likewise able to highlight significant and subtle histological genotypic variations between maize internodes. The grain of precision provided by the workflow also makes it possible to demonstrate different levels of sensitivity to digestion by enzymatic cocktails of the tissues in the pith. The precision and strength of the workflow is all the more impressive because it is preserved on cross section images of other grasses such as miscanthus or sorghum.

**Conclusions:**

The fidelity of this tool and its capacity to automatically identify variations of a large number of histological profiles among different genotypes pave the way for its use to identify genotypes of interest and to study the underlying genetic bases of variations in histological profiles in maize or other species.

**Supplementary Information:**

The online version contains supplementary material available at 10.1186/s13007-022-00957-0.

## Background

Histologically, temperate grass internodes are made of distinct tissues such as vascular bundles, epidermis, rind and pith [1–6]. The localization of each tissue is well defined and two regions are generally physically identified: (i) the rind (outer region) which includes epidermis, sclerenchyma, mesophyll, vascular bundles and parenchyma and (ii) the pith (inner region) which includes parenchyma and bundles [1, 7–10]. Biochemical differences have been reported between rind and pith [2, 3, 8, 10, 11] and a link between tissue type and tissue digestibility has also been reported [1, 2, 8, 12].

The histology of maize stems was also largely revisited recently taking advantages of the development of microscopy, combined with automated image analysis workflows. To resolve histological traits, many strategies have been developed, for example, using a dark background with specific fluorescence filters [5, 13], fluorescence from safranin stained stem sections [14], mass spectrometry imaging [15], X-ray microcomputed tomography [16–20], light background with flatbed document scanner [21], Maüle staining [12, 22], phloroglucinol staining [23, 24], or FASGA (Fucsina, Alcian blue, Safranina, Glicerina and Aqua) staining [12, 25–27].These methods allowed the exploration of large sets of samples. However, the diversity and complexity of histological profiles complicates quantification. The main problems concern the precision of image segmentation and its automation.

Among the different tissue types, bundles in the pith are rather easy to automatically segment and quantify [20, 21, 28]. The use of computed tomography methods even allows to accurately identify bundles in the rind [16–18]. The separation of the rind and the pith is not straightforward and the segmentation between these two regions is often roughly carried out [21, 28]. FASGA staining [29] allows the distinction between pith and rind and the segmentation of lignified and non-lignified tissues in the parenchyma of the pith [4, 12, 25, 28]. Indeed, this stain comprises two dyes: Alcian blue (acidic, anionic dye) and Safranin O (basic, cationic dye stains). As lignins with their phenolic hydroxyl groups are acidic, Safranin O stains highly lignified tissues in red whereas the tissues not or poorly lignified are stained in blue by Alcian blue [29]. Legland et al. [28] developed a workflow that, after parameterization, automatically segments FASGA stained maize cross section images into 4 regions: bundles, rind, lignified parenchyma of the pith and non-lignified parenchyma of the pith. However, parameterization prior to image analysis prevents direct comparison of results between images. In addition, segmentation of tissues within each region is not provided by this workflow.

Herein, we present an ImageJ/Fiji plugin that segments FASGA stained maize internode cross section images by sorting/classifying each pixel according to their H, S and V values and their position in the cross-section image and this without any preliminary parameterization. We also show that the workflow precisely segments the cross section into different tissue types in an automatic and robust manner, and this segmentation reflects (i) the wide variation of tissue types presents in maize internodes and (ii) their ability to be more or less easily digestible when subject to enzymatic attack. In addition, the workflow allows the identification of subtle variations in bundles, pith and rind present between stained cross-section images from different genotypes which no other workflow offers to date. This paves the way to the study of large series of samples and in particular to the description of genetic determinants of anatomical variations in maize stems and the identification of histological bases underlying different biomass end uses such as digestibility of maize silage.

## Results

### An efficient workflow to automatically segment maize internode cross section images into 40 tissue types.

**Fig. 1:**
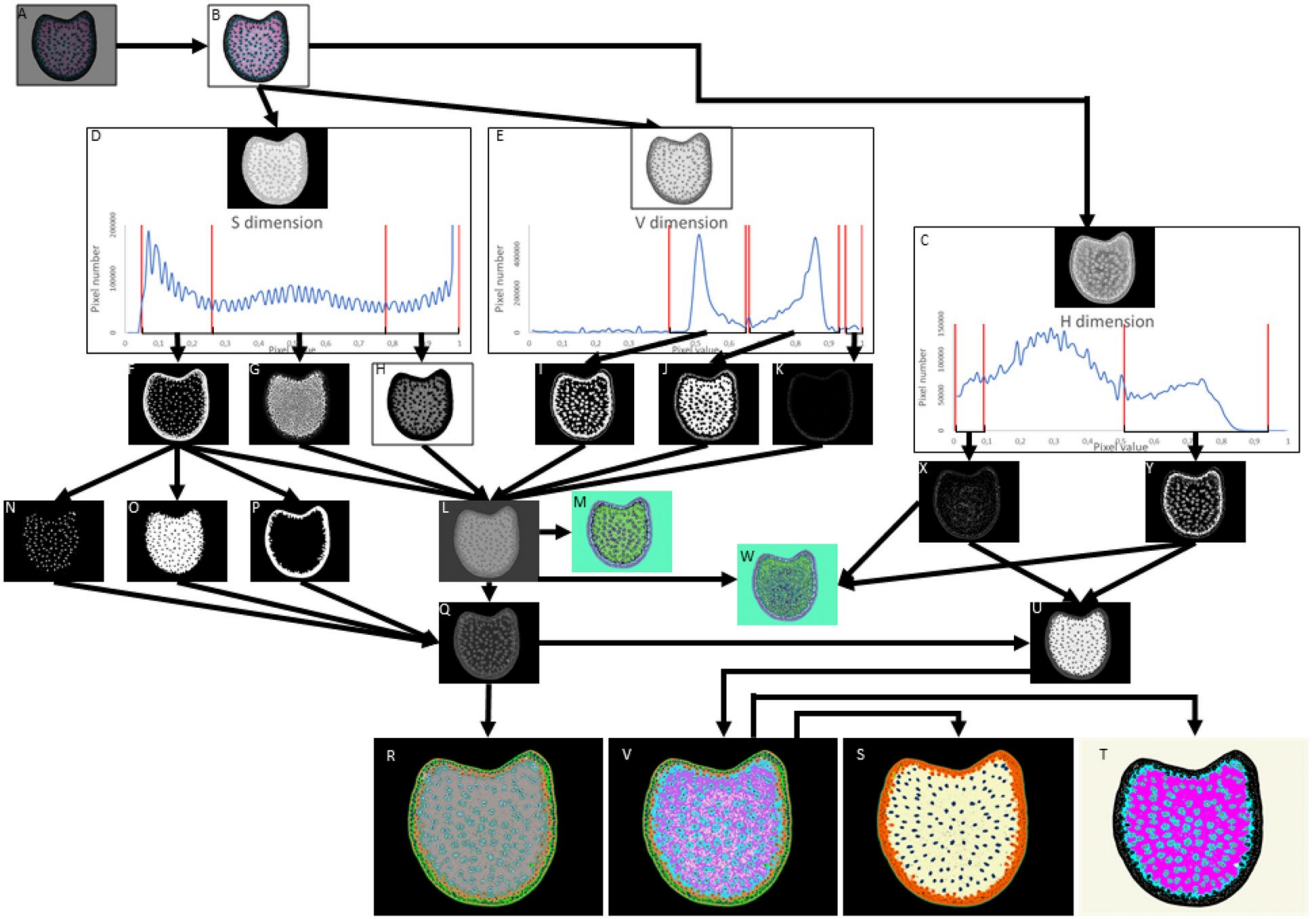
Overview Workflow. **A** Maize FASGA stained internode cross section image. **B** Maize FASGA stained internode cross sections resulting of 3 repetitions of mean radius 2. **C** Image of H dimension and the distribution of the number of pixels according to their H values. **D** Image of S dimension and the distribution of the number of pixels according to their S values **E** Image of V dimension and the distribution of the number of pixels according to their V values **F** Selection of pixels with S value between 0.05 and 0.26. **G** Selection of pixels with S value between 0.26 and 0.78. **H** Selection of pixels with S value between 0.78 and 1. **I** Selection of pixels with V value between 0.42 and 0.65. **J** Selection of pixels with V value between 0.66 and 0.93. **K** Selection of pixels with V value between 0.95 and 1. **L** Filter addition spectrum. **M** Color code image of image presentation in panel L. **N** Density spectrum corresponding to bundles in parenchyma. **O** Density spectrum representing pith and light rind **P** Density spectrum representing dark rind. **Q** Filter addition spectrum with spacial segmentation. **R** FASGA3, Filter addition spectrum with special segmentation colored. **S** Segmentation in 5 histological regions. **T** Segmentation of the tissues of the cross section according to Legland et al. **U** Image segmentation with H option filter colored **V** Image segmentation with H option filter colored. **W** Color code image of image presentation in panel L and including segmentation with H dimension. **X** Selection of pixels according to their H value between 0.005 and 0.09 **Y** Selection of pixels according to their H value between 0.51 and 0.94

FASGA stained maize internode cross section images (Fig. 1B) are converted in H, S and V dimensions and split into H, S and V images (Fig. 1C–E, respectively). The power of this workflow lies primarily in the use of these H, S and V informations. The pixels from image in Fig. 1D are divided according to their S value: pixels with S values between 0.05 and 0.26, between 0.26 and 0.78 and between 0.78 and 1 are extracted in images presented in Fig. 1F–H, respectively. Similarly, 3 ranges of V values (between 0.42 and 0.65, between 0.66 and 0.93 and between 0.95 and 1) enables the extraction of 3 images presented in Fig. 1I–K, respectively. The ranges of variation defined for the S and V values of the pixels have been identified empirically on set of images and are identical regardless of the FASGA stained maize internode cross section image analyzed.

Filtering the pixels according to these ranges of S and V values enables the identification of 15 pixels types (the 6 pixels types according to the S and V values individually and 9 pixels types resulting from the combination of these S and V filters). These 15 pixels types are presented in Fig. 1L and M. The pixel types thus obtained can correspond to different tissues localized either in the rind, in the pith or in the bundles. A spatialization was added in order to combine pixel type and spatial localization in the cross section. To do so, cross section regions (namely the rind, the pith and the bundles) are automatically defined thanks to the use of masks from filtered image with S values between 0.05 and 0.26 (Fig. 1F). These masks enable the separation of tissues in the bundles mask (Fig. 1N), in the mask of dark rind (Fig. 1P) and in the mask including both medullary and rind tissues (Fig. 1O). All pixels, whatever their H, S and V values, located within the dark rind mask (Fig. 1P) are considered to belong to a Dark Rind Tissue (DRT) type or to an Epidermal Tissues type (ET). Pixels in this dark region have been segmented into 9 DRT and to 2 ET according to their S and V values (Fig. 1Q and R and Table 1).

Pixels, whatever their H, S and V values located in the bundle mask (Fig. 1N) belong to a Bundle Tissue (BT) type. These pixels have been segmented into 5 BT types according to their S and V values (Fig. 1Q and R and Table 1). Pixels located in the mask presented in Fig. 1O are located either in Medullary Tissues (MT) or in Rind Tissue considered as Light (LRT). Pixels in this mask have been segmented into 10 LRT and 10 MT type according to their S and V values (Fig. 1Q and R and Table 1). Thus, combining the different pixel types presented in Fig. 1L and the spatial localization of the pixels in the cross section image (Fig. 1N–P) allowed the segmentation of the image into 36 tissue types (Fig. 1Q and R and Table 1).

In addition, the tissues of the different regions of the section are grouped together by the workflow according to two different approaches: (i) according to the tissue belonging to regions of the cross section defined in Fig. 1N–P by distinguishing the tissue types allocated to these regions (Fig. 1S) or (ii) by using the grouping of the tissues of the section as presented in Legland et al. [28] with lignified medullary tissue gathering MT1 and MT4 segmented tissues and low lignified medullary tissue gathering MT2 and MT5 tissues (Fig. 1T).

On the medullary tissues MT1, MT2, MT4 and MT5, the supplemental use of filter values on the H dimension extracted image (Fig. 1C) also allows a more defined segmentation of these tissue types (Fig. 1U–W). For the pixels located in lignified medullary tissues (MT1 and MT4), pixels bearing a H values from 0.005 and 0.09 form the tissue types MT1a and MT4a and the others form the tissues types MT1b and MT4b (Fig. 1X; Table 1). Similarly, for the pixels located in poorly lignified medullary tissues (MT2 and MT5), pixels bearing H values between 0.51 and 0.91 form the tissue types MT2a and MT5b whereas the others pixels form the MT2b and MT5b tissue types (Fig. 1Y; Table 1). In the end, the developed workflow allows the identification of 40 tissue types in FASGA stained maize internode cross section (Fig. 1V; Table 1).

## An easy to use workflow

### Enzymatic digestion on cross sections allowed to clarify the digestibility of the different segmented tissues

**Fig. 2:**
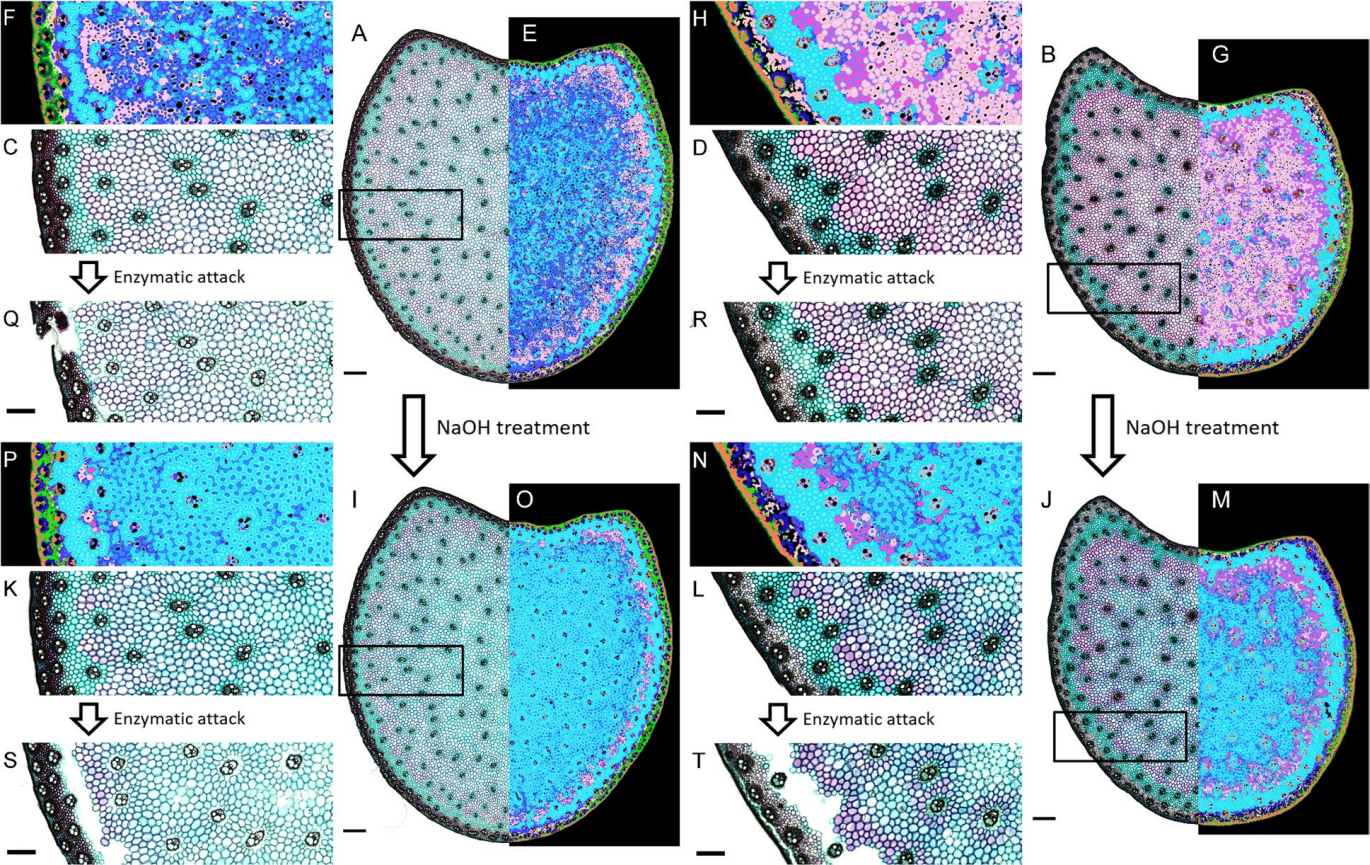
Enzymatic digestion on maize cross section. **A** Maize hybrid FASGA stained internode cross section. **B** Maize inbred line FASGA stained internode cross section. **C** Focus on part of panel A. **D** Focus on part of panel B. **E** Color segmentation images resulting from the proposed workflow on cross section presented in panel A. **F** Color segmentation image resulting from the proposed workflow of panel C. **G** Color segmentation image resulting from the proposed workflow on cross section presented in panel B. **H** Color segmentation image resulting from the proposed workflow of panel D. **I** Maize hybrid internode cross section treated with 0.1 M NAOH solution and stained with FASGA solution. **J** Maize inbred line internode cross sections treated with 0.1 M NAOH solution and stained with FASGA solution. **K** Focus on part of panel I. **L** Focus on part of panel J. **M** Color segmentation images resulting from the proposed method on cross section presented in panel J panel. **N** Color segmentation images resulting from the proposed method of panel L. **O** Color segmentation images resulting from the proposed method on dash point region of cross section presented in panel H panel**.** **P** Color segmentation images resulting from the proposed method of panel I. **Q** Maize hybrid cross sections treated with cellulase solution and stained with FASGA. **R** Maize inbred lines cross sections treated with cellulase solution and stained with FASGA. **S** Maize hybrid cross sections treated with NaOH 0.1 M, cellulase solution and stained with FASGA. **T** Maize inbred lines cross sections treated with NaOH 0.1 M, cellulase solution and stained with FASGA. **Q**, **R**, **S** and **T** Cross section treated with cellulase corresponding to focuses presented in panel C, D, K and L respectively. **A**, **B**, **E**, **G**, **I**, **J**, **O** and **M** Scale bar = 1000 µm. **C**, **D**, **F**, **H**, **K**, **L**, **N**, **P**, **Q**, **R**, **S** and **T** Scale bar = 500 µm.

Two highly contrasting maize internode cross sections, from a hybrid (Fig. 2A) and from an inbred line (Fig. 2B), were selected. The hybrid section exhibits a high bundle density in the rind compared to the cross section from the inbred line (Fig. 2A–D). In addition, the lignified medullary parenchyma (stained in red with FASGA) of the inbred line is more colored than that of the hybrid (Fig. 2A–D), reflecting more lignified cell walls in this tissue in the inbred line. The intensity of the poorly lignified parenchyma (stained in blue with FASGA around the pith) is typically more intense in the inbred line than in the hybrid. The workflow was used to segment the different tissues present within these two internode cross sections stained with FASGA. Within the pith, the lignified medullary tissues (MT1a, MT1b, MT4a and MT4b) are more present in the pith of the inbred line compared to the one of the hybrids (Fig. 2E–H). Note that MT1a and MT4a tissues are almost non-existent in the cross section of the hybrid (Fig. 2E and F). It is found that the tissues that make up the pith of the hybrid are not very lignified compared to those of the inbred line.

The impact of 0.1 M NaOH treatment for one hour resulted in cell wall modifications reflected by post-treatment FASGA color modifications. Notably, the lignified medullary tissues turn blue in both the hybrid and the inbred line (Fig. 2I–L). The images of sections treated with NaOH and stained with FASGA were analyzed with the workflow and the output of this analysis clearly underlines a bluing of the tissues in the pith with in particular a predominance of the appearance of tissue type MT2b and MT5b in the inbred line (Fig. 2M and N) and in the hybrid (Fig. 2O and P).

Enzymatic digestion on a non NaOH treated cross section (Fig. 2Q and R) for one hour also underlines differences in the behavior of the tissues within a section but also differences between the hybrid (Fig. 2Q) and the inbred line (Fig. 2R). In the inbred line (Fig. 2R), the blue staining with FASGA after enzymatic treatment shows a reduced intensity of blue for the lowly lignified pith tissues at the periphery of the pith (MT2b, MT5b, Fig. 2H and Table 1). In the hybrid, these lowly lignified pith tissues were digested (Fig. 2Q) while the MT2a and MT5a tissues located mainly in the center of the parenchyma in the hybrid were not fully digested during the enzymatic treatment. This highlights the higher sensitivity to enzymatic digestion of tissues TM2b and 5b compared to TM2a and 5a.

0.1 NaOH treated cross sections were also subjected to enzymatic digestion. In the inbred line, the poorly lignified MT2b and MT5b tissues at the periphery of the pith were fully digested (Fig. 2H, N and T). The initially lignified tissues (MT1b and MT4b) turned blue as during the NaOH treatment without enzymatic digestion, but also had a reduced blue intensity, underlining a partial digestibility of these tissues (Fig. 2F, P, S, H, N and T). The initially lignified MT1a and MT4a medullary tissues turned blue as during the NaOH treatment alone, but showed no signs of digestibility (Fig. 2H, N and T). In the hybrid, the NaOH treatment followed by an enzymatic digestion resulted in a more substantial bluing over the entire surface of the section with a high proportion of medullary tissue digested at the periphery and in the center (Fig. 2I, K, and S), corroborating as for the inbred line with a strong presence MT2b and MT5b tissues (Fig. 2O and P).

Segmentation of these treated and untreated cross sections has been carried out using the proposed workflow without further parametrizations, allowing us to automatically highlight histological differences between the selected hybrid and inbred line but also between treatments. This analysis also revealed the high sensitivity to enzymatic digestion of TM2b and TM5b tissues.

### The workflow is able to highlight significant and subtle histological variations between maize cross section images

**Fig. 3:**
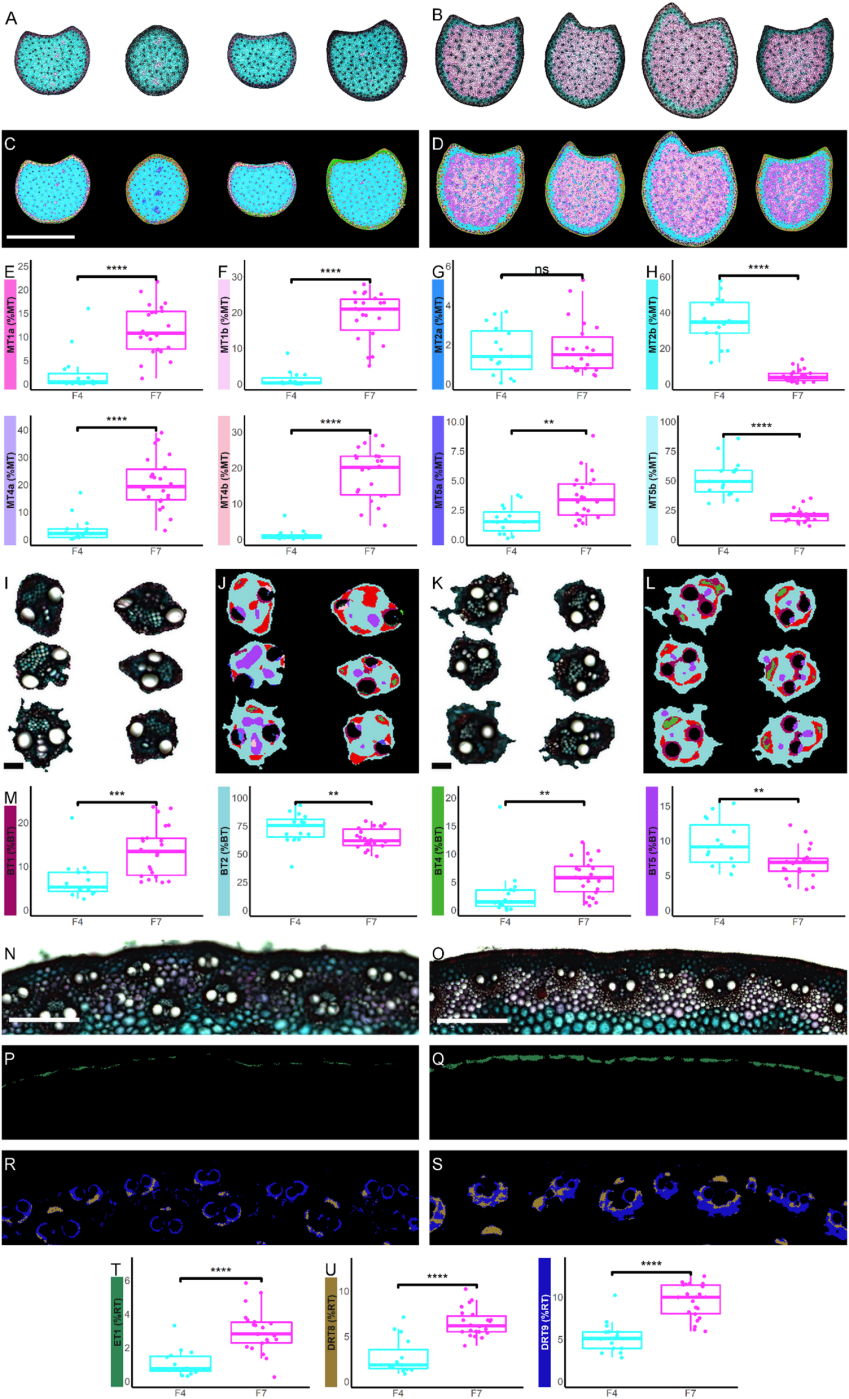
Result of the proposed workflow on F4 and F7 maize inbred lines cross section FASGA stained images. **A** F4 inbred line FASGA stained internode cross sections images. **B** F7 inbred line FASGA stained internode cross sections images. **C** F4 segmented images resulting from the proposed workflow. **D** F7 segmented images resulting from the proposed workflow. **E** Quantified area of lignified parenchyma tissues MT1a and MT4a (on top and bottom, respectively) from F4 (in blue) and F7 (in pink) cross section, expressed in percentage of medullary tissues (%MT). **F** Quantified area of lignified parenchyma tissues MT1b and MT4b (on top and bottom, respectively) from F4 (in blue) and F7 (in pink) cross section, expressed in percentage of medullary tissues (%MT). **G** Quantified area of low lignified parenchyma tissues MT2a and MT5a (on top and bottom, respectively) from F4 (in blue) and F7 (in pink) cross section, expressed in percentage of medullary tissues (%MT). **H** Quantified area low lignified parenchyma tissues MT2b and MT5b (on top and bottom, respectively) from F4 (in blue) and F7 (in pink) cross section, expressed in percentage of medullary tissues (%MT). **I** Magnifications of bundles from F4 inbred line FASGA stained cross section images obtained in panel A. **J** Segmented images of F4 inbred lines bundles. **K** Magnification of bundles from F7 inbred line FASGA stained cross section images obtained in panel B. **L** Segmented images of F7 inbred line bundles. **M** Quantified area of bundle tissues (BT1, BT4, BT2 and BT5) from F4 (in blue) and F7 (in pink) cross section, expressed in percentage of bundle tissues (%BT). **N** Magnification of rind region of F4 inbred lines FASGA stained cross section image. **O** Magnification of rind region of F7 inbred lines FASGA stained cross section image. **P** F4 ET1 segmented images tissues from the region presented in panel N. **Q** F7 ET1 segmented tissues from the region presented in panel O. **R** F4 DRT8 and DRT9 segmented tissues from region magnified in panel N. **S** F7 DRT8 and DRT9 segmented tissues from region magnified in panel O. **T** Quantified sclerenchyma hypodermis tissues ET1 from F4 (in blue) and F7 (in pink) cross section, expressed in percentage of rind tissues (%RT). **U** Quantified area of sclerenchyma tissues DRT8 and DRT9 (on the left and on the right, respectively) from F4 (in blue) and F7 (in pink) cross section, expressed in percentage of rind tissues (%RT). Significance: no symbol, p ≤ 0.1; *, p ≤ 0.05; **, p ≤ 0.01; ***, p ≤ 0.001; ****, p ≤ 0.0001. **A**, **B**, **C** and **D** Scale bar = 1 cm. **I**, **J**, **K** and **L** Scale bar = 100 µm. **N**, **O**, **P**, **Q**, **R** and **S** Scale bar = 500 µm

It has been reported in the literature that F4 maize inbred line presented a very poorly lignified pith [12, 26, 30, 31]. This result is again confirmed in our study (Fig. 3A), with F4 showing a pith stained in blue with FASGA. Conversely, the F7 maize inbred line presented a histological profile more typical of a maize inbred line, with a predominantly red parenchyma in the pith and with the tissues at the periphery of the pith stained in blue (Fig. 3B). Again, the use of the workflow captures these obvious differences at the cross section level (Fig. 3C and D).The quantification of the area of the different tissue types in the workflow clearly indicates that the internodes from F7 line had significantly more MT1a, MT4a, MT1b and MT4b tissues in its pith (Fig. 3E and F) while the internodes from F4 line had significantly more MT2b and MT5b (Fig. 3H). On the other hand, the workflow indicates that the F7 line has more MT5a tissues (tissue stained blue by FASGA) than the F4 line (Fig. 3G). This suggests that the pith of the F4 line is overwhelmingly composed of a non-lignified parenchyma that is very easily digestible.

The workflow is also able to highlight the differences in the presence of different tissues within the bundles, these differences being very difficult to identify with the naked eye. The workflow demonstrated that the F7 line has significantly more BT1 and BT4 tissues than the F4 line (Fig. 3M). BT1 and BT4 tissues are localized at the sclerenchyma poles of the bundles with BT1 surrounding and also connecting the meta-xylem bundles forming the xylem fiber (Fig. 3I–L). BT1 and BT4 tissues are therefore strongly lignified. In contrast, the F4 line possessed significantly more BT2 and BT5 tissues than the F7 line (Fig. 3M). BT2 is the most extensive tissue in the bundles and represents the bundle parenchyma which sometimes extends over the first parenchymal cell layer surrounding the bundle (Fig. 3I–L). Finally, BT5 represents the phloem of bundles. BT2 and BT5 are therefore poorly lignified tissues (Fig. 3I–L). F4 therefore has bundles composed more predominantly of easily digestible tissues than the F7 line.

Along with the rind, the epidermis of F7 has significantly more ET1 tissue (Fig. 3N, O, P, Q and T). Remarkably, the workflow was also able to point out that F7 had more DRT8 and DRT9 tissue types (Fig. 3R, S and U). These two tissues are included in the dark rind region and specifically represent schlerenchyma of bundles located in the rind. Thus, the F7 line has more schlerenchymatous bundles in the rind and in the pith. The high level of degradability of F4 may come from the fact that it has a parenchyma in the pith that is poorly lignified (as reported in [12, 26, 31]) but the fact that its bundles are less schlerenchymatous can also support strong degradability of F4. It is essential to reach this level of segmentation of the cross section images in order to be able to demonstrate these histological differences. The proposed workflow is capable of achieving this level of precision.

### Universal segmentation workflow

**Fig. 4:**
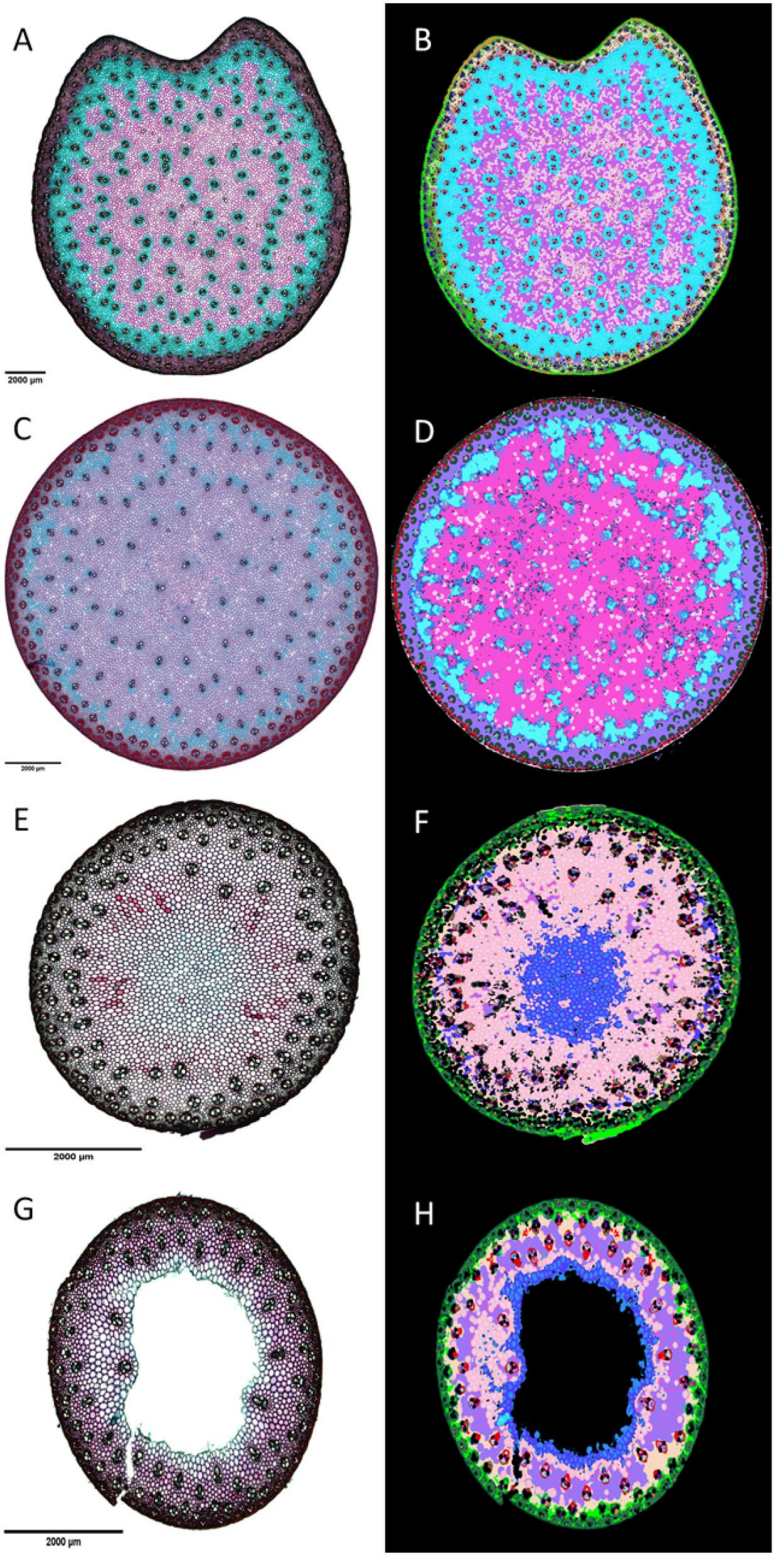
Result of the proposed workflow on internodes cross section FASGA stained images from different grass species. **A** Maize FASGA stained internode cross section. **B** Color segmentation of image A resulting from the proposed method. **C** Sorghum FASGA stained internode cross section. **D** Color segmentation of image **C** resulting from the proposed method. **E** and **G** Miscanthus FASGA stained internode cross sections. **F** and **H** Color segmentation of images **E** and **G** resulting from the proposed method

The universality of the presented workflow has been proven using images of stem sections of different grasses such as maize, sorghum and miscanthus (Fig. 4). The workflow has also been challenged on lower quality cross sections (Additional File 2) and thus demonstrated its ability to segment faithfully the image even in the presence of holes. The stems of grasses show a fairly similar tissue organization overall, with a peripheral region (rind) dense in vascular bundles surrounded by schlerenchyma and a medulla region (pith) with a lower bundle density [32].

The FASGA-stained sorghum internode section image (Fig. 4C) was produced by Christelle Baptiste, David Pot and Jean-Luc Verdeil (CIRAD, Montpellier) and shows a more distinct FASGA staining, due to a different section thickness and/or a FASGA staining protocol that differs slightly from the one used for maize internode cross sections (Fig. 4A and C). Anyway, the use of the workflow allowed a quality segmentation of the image of the sorghum cross section (Fig. 4D). Perrier et al. [4] proposed an image analysis method to segment sections of sorghum internodes stained with FASGA. These authors point to the presence of a rind and a pith which they call zone Z1 and Z2 respectively. The herein proposed workflow also pinpointed these two regions with a more detailed segmentation, as in the case of maize internodes (Fig. 4B and D). Interestingly, using our workflow, precise segmentation of the blue FASGA stained parenchyma located in the peripheral of the pith was carried out. In addition, using the H value of pixels of this tissue (Fig. 1) allowed us to also distinguish different types of tissues in pith parenchyma (Fig. 2). The sorghum blue FASGA stained parenchyma surrounding the pith region has been segmented into MT2b and MT5b, suggesting the fact that this tissue is potentially easily digestible, as in the case of maize internode (Fig. 4B).

Miscanthus internodes presented in Fig. 4E and G have been harvested in February, at a much more mature stage than the one of maize and sorghum presented in Fig. 4. Cell wall proprieties evolve with internode maturity [33], notably by increasing lignification and decreasing digestibility of internode tissues. A comparison between maize and miscanthus internode at the biochemical and histological level was previously presented [34]. The protocol used (cutting thickness, staining and image acquisition) to characterize miscanthus internodes is strictly the same as the one used to characterize the maize internodes presented in this study. FAGSA stained miscanthus internode cross section (Fig. 4E and G) showed differences in staining compared with the ones of maize and sorghum (Fig. 4A and 4C). The image analysis workflow proposed was able to highlight these differences of staining both in rind and pith regions (Fig. 4F and H). In accordance with the fact that overwinter miscanthus internode are less digestible than silage stage maize or sorghum internodes, tissues identified in pith of miscanthus predominantly belong to the MT1b types. Parenchyma located in the center of the pith region is FASGA stained in blue and the workflow identified MT2a and MT5a tissue types in this region. In the case of the presence of a medullary hole, tissues surrounding this hole are also of MT2a and MT5a type.

The workflow was then able to distinguish different types from FASGA stained images of stems from different grass species. This open opportunity to highlight histological differences between species, between different stage and different internode levels.

## Discussion

### A faithful and automatic workflow that can be used on different grass species

The internode of maize is made up of different tissue types [7]. These tissues are precisely distributed in different regions of the internode. So, there are tissues in and around the bundles that are distributed loosely in the medullary region and more densely in the rind region. The medullary region is mainly comprised of parenchyma [2]. The FASGA staining highlights parenchyma colored in red, signing lignified cell walls while some parenchyma are colored blue, signing low lignified parenchyma in maize [12, 25] and in sorghum [4] and in miscanthus [34]. In particular, this staining makes it possible to highlight in maize lowly lignified cell walls of the parenchyma located on the periphery of the parenchyma and near the rind and also sometimes the cell walls around the bundles [6, 25]. These poorly lignified regions coincide with the easily enzymatically digestible regions [2]. In the rind, beyond the presence of bundles, parenchyma (lignified or poorly lignified revealed by different types of staining including FASGA) and sclerenchyma are present. The rind is surrounded by cell layers that make up the epidermis.

Different cross section image analysis workflows allow to distinguish the presence of the different tissue types described above. Briefly, on freshly harvested and manually truncated internodes, Heckwolf et al. [21] offers a workflow that roughly quantifies the thickness of the rind as well as the presence (number and area) of bundles in the pith. The rind is delimited by considering a visual difference in texture between the outer and inner part of the cross section. Zhang et al. [25] workflow uses for the first time FASGA stained cross section images and accounts for variation in lignification in different tissue types (between rind and pith but also within the pith). Du et al. [16] and Zhang et al. [20] proposed to segment an internode cross section image using micro-computed tomography. These authors focused mainly on the bundles by quantifying parameters relating to their number, area and geometry. Remarkably, Zhang et al. [20] also proposed to segment precisely the cross section into an inner zone (pith), a periphery zone (rind) and an epidermis zone. FASGA staining has also made it possible to segment maize internode cross section images [28, 35] or sorghum images [4]. This allows in particular to roughly delimit the pith of the rind and to offer workflows that automatically separate lignified medullary tissues from poorly lignified medullary tissues. Segmentation using the presented workflow herein makes it possible to separate 40 tissue types within a maize internode cross section by allocating them to different regions of the cross section (epidermis, dark rind, light rind, lignified medullary, low lignified medullary tissues and bundles). This exhaustive segmentation is the result of a characterization of the pixels according to their H, S and V values and their location in the cross section. Corcel et al. [13] notably report multispectral characteristics specific to each tissue type in maize internode cross section. The proposed workflow faithfully reflects the tissue diversity present in maize internode. Furthermore, this workflow reaches a satisfactory level of precision for separating different regions of the cross section as proposed by Zhang et al. [20]. It points in particular to different cell types in these different regions, which is an improvement compared to the workflow proposed by Legland et al. [28] and brings a great level of precision to tissue identification within each region. Tomography remains the technique of choice for individualizing bundles in the rind [16], but the proposed workflow still allows the identification of many different tissue types in this region. The important advantage to note with the proposed workflow is that it is completely automatable and does not require any parameterization for all the cross-section images. This makes it possible to avoid workflow quantification differences due to a parameterization of each image, which is not the case with Perrier et al. [4] or in Legland et al. [28]. In addition, the fact that the proposed workflow is usable on different grass species allows the comparison of the tissues between internodes from different species, including grasses with hollow stems such as miscanthus and also rice, wheat, brachypodium, etc.

In conclusion, the developed workflow enables users to efficiently analyze FASGA stained grass internode cross section images in an automated manner (i.e. without manual intervention/or parameter tuning), providing plant scientists with a powerful and precise analytical tool that produces reliable quantitative data.

### An exhaustive segmentation which faithfully reflects variations in tissue type

Enzymatic attack on sections of maize internodes not treated with 0.1 M NaOH only digests non-lignified pith tissue, especially on the periphery of the pith, around the bundles and the phloem in the bundles. Similar observations have already been reported using rumen juice to digest sections of maize internodes [2, 8, 36–40]. The surfaces of the digested section coincide with the presence of poorly lignified tissues colored blue by FASGA and segmented precisely by the proposed workflow (MT2b, MT5b, BT2 and BT5), emphasizing the fact that the segmentation of the workflow is relevant. In particular, we note the presence of medullary tissues that are not very lignified (colored blue with FASGA) but which remain poorly digested during the enzymatic attack. These tissues are identified and segmented by the workflow (MT2a and MT5a). In addition, the use of an NaOH treatment made it possible to break the ester bonds and thus released the esterified ferulic acids and diferulic acids (involved in the bonds between hemicelluloses chains; [41–46]) and the esterified *para*-coumaric acids (mainly present in the cell wall of grasses [33, 46];). These modifications caused a relaxation of the parietal mesh and facilitate the accessibility of enzymes to the wall [38, 47]. The 0.1 M NaOH treatment increased the surface area of MT2b and MT5b tissues within the pith, corroborating with the increase in digestibility after this treatment. This indicates that the FASGA staining highlighted the wall modifications generated by the NaOH treatment and that the workflow is sensitive to identify these modifications. Taken together, these results underscored that the differences in segmented tissue within the pith reflect significant biochemical variations in tissue type.

In conclusion, the fine and precise segmentation reflects variation between tissue types. Some of the properties are evidenced with the variation in the ease of being enzymatically digested. The level of precision provided by the workflow translates a biological reality.

Overall and again, the presented workflow allowed to faithfully and automatically segmented FASGA stained maize internode cross section, allowing the identification of variation (even subtle) between internodes from different genotypes. This underlies the fact that this analytical tool is therefore efficient and ideal for its use in selection and/or genetics studies [19, 27, 48]. Indeed, using this workflow allowed us to pinpoint obvious variation between histological profiles of F4 and F7 previously reported (Barrière et al., 2017) but it also allowed us to highlight more subtle variations such as the presence of more schlerenchymatous tissues of F7 bundles compared to the bundles of F4, corroborating also the fact that F4 is more easily degradable than F7.

## Conclusions

Among the set of workflows developed to characterize histological profiles of maize internodes the workflow proposed herein provides a precise level of segmentation within the entire section by segmenting 40 tissue types including: 2 tissues in the epidermis, 19 tissues in the rind, 14 tissues in the pith and 5 tissues in the bundles. This level of segmentation is achieved by combining both the FASGA staining, the H, S and V properties of each pixel and the location of each pixel in the cross section. This level of segmentation reflects differences in the cell wall properties of each tissue. In particular, the variation in the ability to be easily digested is accounted for by the segmentation of tissues in the pith. The fidelity of this tool, its capacity to automatically identify variations of a large number of histological profiles among different genotypes pave the way for its use to identify genotypes of interest and to study the underlying genetic bases of variations in histological profiles in maize.

## Methods

### Plant materials

F4 and F7 inbred lines were cultivated in a field trial in Versailles in 2020 in two 3 m rows with 0.50 m between rows. At silage stage, two internodes under the main ear from two plants of a row were cut and stored in 70% ethanol for further histological analyses. Internodes from a hybrid were harvested from plants cultivated in open field trials in summer 2019 at Villampuy in two 7 m rows with 0.80 m between rows. At silage stage, internodes below the main ear from two plants were cut and stored in 70% ethanol for further histological analyses.

### Cross section staining and image acquisition

Similar to Legland et al. [28], internode cross section of 150 µm were cut at one centimeter below the upper node with a microtome [49] and stained with FASGA solution (for more details of the staining proptocol, see Legland et al. [28] or El Hage et al. [26]). Briefly, the cross sections were soaked in FASGA solution diluted in distilled water (1:8, v/v) for 24 h under agitation and then rinsed with distilled water for 24 h under agitation. Images of stained stem cross-sections with resolution of 5.17 μm per pixel were acquired (an example is presented in Fig. 1A) using the same procedure as the one described in Legland et al. [28] (slide scanner piloted by the Metafer scanning, imaging platform (MetaSystems GmbH, Altlussheim, Germany)).

### Image segmentation

Before being able to use the segmentation workflow presented in this paper it is necessary to load the plugin colortransform in order to be able to convert the RGB coded image pixels in another color-coding system (in our case, to convert pixels into HSV coded pixels).

The automatic segmentation imaging workflow described was developed using the ImageJ/Fiji plateform [51], using H, S and V segmentation [50]. The whole workflow is freely downloadable and easily implementable as an ImageJ/Fiji plugin (https://drive.google.com/file/d/1jH2GQmNA9QNL1XqWtdGrIhgDIY0xLNc7/view?usp=sharing). After loading the plugin developed in the dedicated directory of Fiji, you simply need to open it and to answer 8 questions (Additional File 1) to define the desired output data set. Prior to this, you are asked which directory to use to load the input images and in which directory to put the output images and the results tables.

The different steps of the workflow are described in the results section. A multiplication by 2 of RGB pixel values and a Gaussian smoothing was applied to increase contrast and reduce acquisition noise, respectively (Fig. 1B). The images obtained were transformed from Red, Green and Blue (RGB) channels to H, S and V dimensions using a color transform workflow [50]. Three images were extracted according to their H, S and V values (Fig. 1C, D and E). Three ranges of S ([0.05:0.26]; [0.26:0.78]; [0.78:1]) and V ([0.42:0.65]; [0.66:0.93]; [0.95:1]) values were empirically defined in order to better segment images using the following steps. Images with pixel S or V values included in each range mentioned above were extracted (Fig. 1F, G and H for the 3 ranges of S values and 1I, 1 J and 1 K for the 3 ranges of V values). Combining pixels from these different filters allowed the identification of 15 pixel types (the 6 pixel types according to the S and V values individually and 9 pixel types resulting from the combination of these S and V filters; Fig. 1L and M). In order to segment the images at the level of tissue types, masks identifying cross section regions (the rind; bundles and the pith including partially the rind) were defined using filtered pixels with S values between 0.05 and 0.26 (Fig. 1N, O and P for the 3 masks). Combining the different pixel types with their position in a mask region lead to the identification of 36 tissue types (Fig. 1Q and R). On the tissues belonging to the pith masks, the use of filter values on the H dimension extracted image (Fig. 1C), also allowed a more defined segmentation of these tissue types (Fig. 1U–W). At the end, 40 tissue types are segmented from a FASGA stained cross section image using this workflow. H, S and V values used to filter the images and to produce masks do not depend on the analyzed image allowing automatization of the workflow.

### Enzymatic digestion of internode cross sections

Ten successive internode cross sections were used to test two different digestion procedures followed by FASGA staining in order to use the developed workflow. The first digestion procedure is the application of 3 mL of a cellulase solution 1 g/L (« Onozuka R-10 from *Trichoderma viride*»; Serva; 10 mg ref 16,419.03) at 40 °C on previously water rinsed cross sections (H_2_0 during 3 h at room temperature). The second digestion procedure is the application of the cellulase solution mentioned above 1 g/L 3 mL at 40 °C on previously NaOH (0.1 M) treated cross sections during 1 h and rinsed (H_2_0 during 3 h at room temperature). NaOH (0.1 M) treated and rinsed (H2O during 3 h) at room temperature cross sections were also FASGA stained and analyzed using the workflow.

### Statistical analyses

Wilcoxon test were carried out using R package ggplot2 function stat_summary [52] to highlight significant differences between F4 and F7 lines. Correlation analyses were carried out using stats R package [53]

**Table 1:**
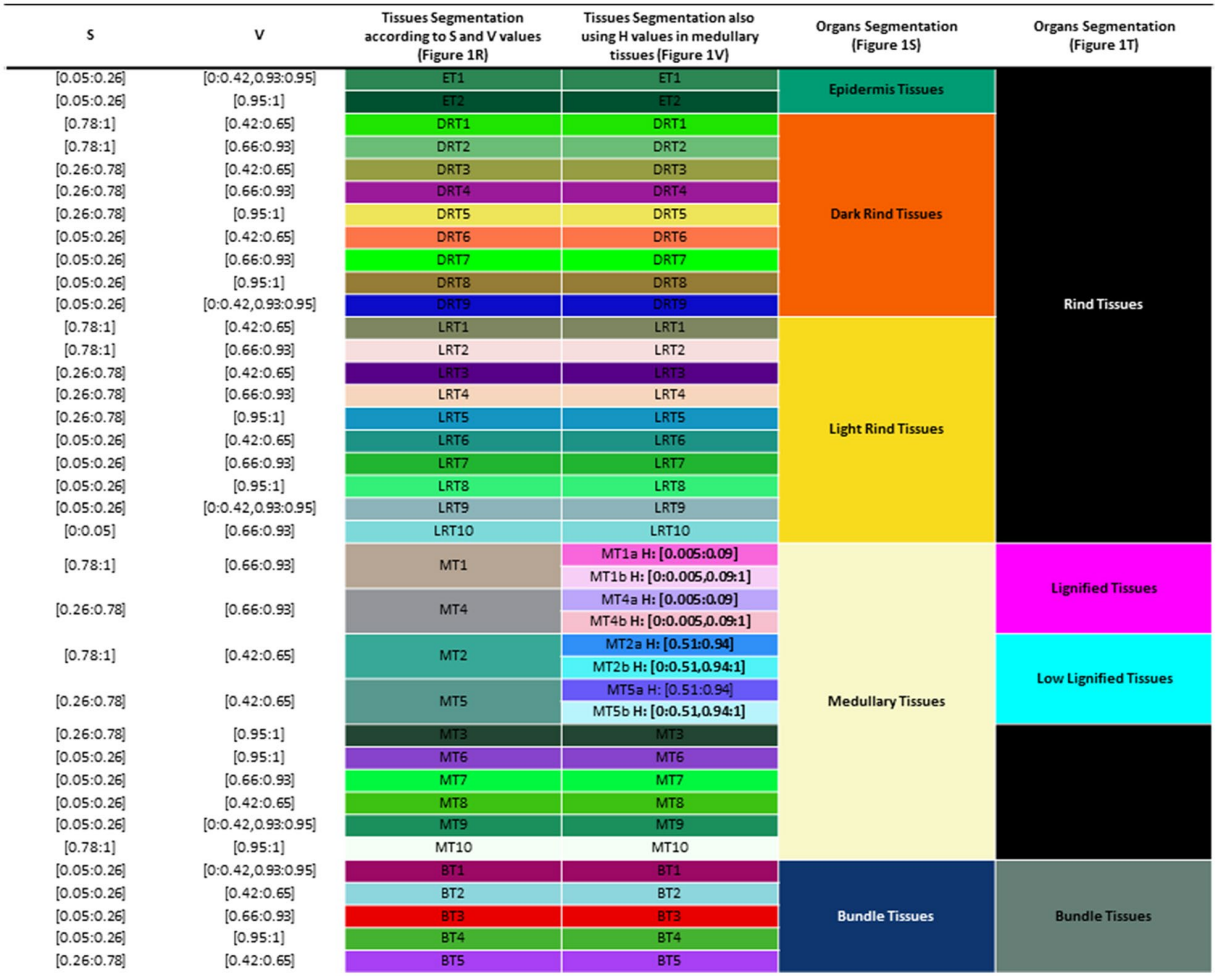
H, S and V parameters of the 40 segmented tissues

H, S and V parameters of the obtained 40 tissues segmented from maize FASGA stained cross section images. Attributed color of each segmented tissue is also reported.

## Supplementary Information

**Additional file 1.** Eight questions asked to define the desired output data set.

**Additional file 2.** The workflow presented in this article also works on images of lower quality. **A:** example of poor quality image used as input to the workflow and **B:** associated output image.

## Data Availability

The datasets used and/or analyzed during the current study are available from the corresponding author on reasonable request.

## Abbreviations

FASGA: Fucsina, Alcian blue, Safranina, Glicerina and Aqua
H: Hue
S: Saturation
V: Value
DRT: Dark rind tissue
ET: Epidermal tissues
BT: Bundle tissue
MT: Medullary tissues
LRT: Light rind tissue
m: Meter
h: Hour
