# Supplementary material for: A robust and efficient automatic method to segment maize FASGA stained stem cross section images to accurately quantify histological profile
Source: Plant Methods. 2022 Nov 24;18:125. doi: 10.1186/s13007-022-00957-0 (PMC9694518; doi:10.1186/s13007-022-00957-0)
Supplement: Supplementary file 1 — Additional file 1. Eight questions asked to define the desired output data set. [file 13007_2022_957_MOESM1_ESM.docx]

**Additional file 1**

The eight questions, to be answered "yes" or "no", are the following ones:

1/ Do you want to export the H, S and V values of the processed images?

By answering "yes" to this question, a “.csv” table quantifying the distribution of pixels in H, S and V classes will be provided. Note that each H, S, V values will be evenly divided into 100 classes.

If you want to select different H, S and V values more adapted to your image, you must modified the values in the workflow in lines :

For S [610, 619, 628, 870, 879, 888, 1040, 1049, 1058, 1427, 2990, 2999, 3008, 3248, 3257, 3266, 3418, 3427, 3436 and 3661].

For V [641, 650, 659, 901, 910, 919, 1071, 1080, 1089, 3021, 3030, 3039, 3279, 3288, 3297, 3449, 3458 and 3467].

For H [4190 and 4218].

2/ Do you want to export results from segmentation of your images?

If you answer "no" to this question, none of the following questions will be of interest and only the output related to question 1 (see above) will be provided. Answering "yes" to this question will then allow you to select the types of exported results by answering questions 3 to 8.

3/ Do you want to clean the background noise of your images?

It is important to use good quality input images with a clean background to perform quality segmentation and quantification. If the input images are already of good quality and/or free of background noise then choose "no" to reduce the time of the process and the number of images generated.

4/ Do you want to segment pixels of your images also according to H values, in addition to S and V values?

The use of the H dimension makes it possible to specify the segmentation in the pith of the TM1, TM2, TM4 and TM5 tissues (Figure 1). Thus, by answering "yes" to this question, the result of the segmentation corresponds to figure 1V and by answering "no" to figure 1R and 1M.

5/ Do you want outputs of this segmentation?

By answering "yes" to this question, a “.csv” quantification table of segmentation results and a segmented image output will be provided to the user.

For instance, if segmentation according to H, S and V values is requested in question 4, a “.csv” format table including 440 columns will be provided. These columns include information according to the surface area (in pixels) of each of the 40 tissues, the 4 TM1, 2, 4 and 5 tissues without distinction of "a" and "b", the 5 synthetic tissues (epidermis, dark rind, light rind, medullary and bundle tissues), the 3 synthetic tissues (rind, lignified and low lignified tissues) characterized by the macro developed by Legland et al. (2017), the total surface area of the cross section, the total surface area of the cross section subtracted from the holes and the surface area of the holes present on the cross section image. For these 55 segmented tissues, the results table also includes the number of counts and the average intensities of H, S, V, R, G and B of each segmented tissue. Each of the 55 tissues is thus characterized by 8 variables.

6/ Do you want an additional table presenting the areas (in pixels) of the segmented image in pixel classes?

By answering "yes" to this question, an additional “.csv” format table will be exported. If the segmentation is performed on the basis of the dimensions S and V, this table will contain the surfaces of 15 classes of pixels (figure 1M) whereas it will contain 45 classes if the segmentation is performed on the basis of the 3 dimensions H, S and V (figure 1W).

7/ Do you want additional image outputs of each segmented tissues types?

If you answer "yes" to this question, output images will be created. Each image corresponds to the location of a specific segmented tissue and is named with the name of the corresponding tissue in the output “.csv” table file (question 5).

8/ Do you want additional image outputs of each segmented pixel types?

If you answer "yes" to this question, output images will be created. Each image corresponds to the location of a specific segmented pixel type and is named with the name of the corresponding tissue in the output “.csv” table file (question 5).
