# Supplementary material for: A robust and efficient automatic method to segment maize FASGA stained stem cross section images to accurately quantify histological profile
Source: Plant Methods. 2022 Nov 24;18:125. doi: 10.1186/s13007-022-00957-0 (PMC9694518; doi:10.1186/s13007-022-00957-0)
Supplement: Supplementary file 2 — Additional file 2. The workflow presented in this article also works on images of lower quality. A: example of poor quality image used as input to the workflow and B: associated output image. [file 13007_2022_957_MOESM2_ESM.docx]

Additional file 2

B

A


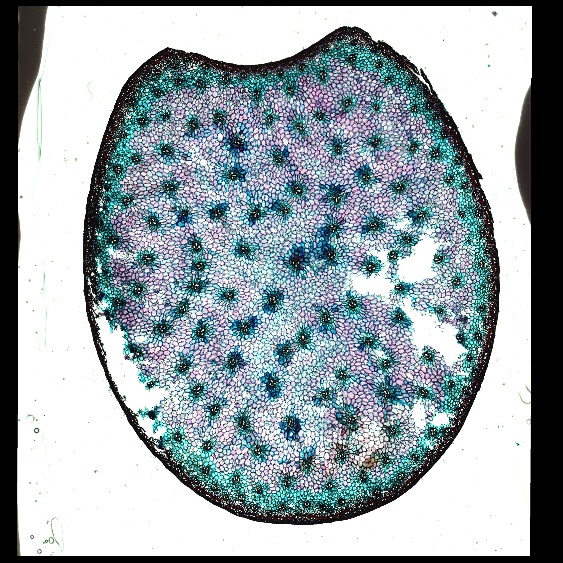




A : Lower quality fasga stained cross section.

B : workflow out put of the segmented image.
